# Mental well-being and test anxiety among students preparing for the university admission exam during the pandemic

**DOI:** 10.3389/fpsyg.2023.1184788

**Published:** 2023-07-21

**Authors:** Eleyza Yatkin, Neriman Aral, Lugen Ceren Gunes, Selim Tosun

**Affiliations:** ^1^Independent Researcher, Zonguldak, Türkiye; ^2^Department of Child Development, Faculty of Health Sciences, Ankara University, Ankara, Türkiye; ^3^Department of Child Development, Faculty of Health Sciences, Graduate School of Health Sciences, Ankara University, Ankara, Türkiye

**Keywords:** distance education, high school students, mental well-being, pandemic, test anxiety

## Abstract

**Objective:**

The present study attempted to explore any potential association between test anxiety and mental well-being among high school students preparing for the university admission exam in times of the pandemic.

**Methods:**

The sample of this correlational study consisted of 427 senior high school students in Caycuma district of Zonguldak, Turkey. The data were collected online using a demographic information form, the Warwick-Edinburgh Mental Well-Being Scale, and the Westside Test Anxiety Scale between April–May 2021.

**Results:**

Our findings revealed student gender, paternal education, availability of a personal room and computer, and motivation for online classes to be factors associated with test anxiety. Besides, we discovered parental age, maternal education and employment, the device used for online classes, perceived effectiveness of distance education, and motivation for online classes to be linked with mental well-being among students.

**Conclusion:**

In a nutshell, several factors were discovered to contribute to test anxiety, including student gender, paternal education, availability of a personal room and computer, and motivation for online classes. The findings also suggested some noteworthy factors influencing students’ mental well-being, such as parental age, maternal education and employment, the device used for online classes, perceived effectiveness of distance education, and motivation for online classes. Finally, we uncovered a significant negative association between the participating students’ test anxiety and mental well-being.

## 1. Introduction

Spreading unprecedently and leading to the demise of thousands, the novel type of coronavirus (COVID-19) is now described as one of the greatest pandemics ever by the World Health Organization (WHO; Akdag, 2020; Tongar, 2020; WHO, 2020). Along with extraordinary social, cultural, and economic processes worldwide (Karatas, 2020), the concept of a “new normal,” mandated by the pandemic, has introduced public transport restrictions, social isolation, quarantine, and curfews, and many schools had to remain closed for a long time (Karatas, 2020; Gilliam et al., 2021). In this process, UNESCO emphasized the inevitableness of distance education since school lockdowns have brought adverse impacts on students and other stakeholders of education (UNESCO, 2020; UNICEF, 2020). In this regard, recent research documented that the pandemic has had impacts on the mental health of adolescents, accompanied by symptoms such as anxiety, stress, panic attacks, irrational anger, impulsivity, somatization disorder, sleep disorders, emotional disturbance, post-traumatic stress symptoms, and suicidal behaviors (Hossain et al., 2020; Chamberlain et al., 2021; Gambin et al., 2021; Magson et al., 2021; Meherali et al., 2021; Sher, 2021).

COVID-19 is not only linked to psychiatric symptoms but also to a range of neurological disorders [e.g., encephalopathy, encephalitis, stroke, seizures, anosmia, psychosis, brain fog, and headache (Achar and Ghosh, 2020)]. For example, the virus can infiltrate the central nervous system (CNS) through possible pathways (e.g., olfactory nerve, hematogenous spread (through the bloodstream), and retrograde axonal transport) and lead to neurological issues (Achar and Ghosh, 2020; McQuaid et al., 2021). Encephalopathy and stroke are the most reported neurological symptoms of COVID-19 (Cervantes-Arslanian et al., 2022; Singh et al., 2022). These symptoms were associated with worse outcomes, including a higher likelihood of intensive care, death, and long-term symptoms. And neurological impacts of COVID-19 are believed to be caused by multiple mechanisms. One mechanism involves the effect of the SARS-CoV-2 virus on blood vessels and blood flow, which can indirectly harm the brain. Another key factor is the disruption of the immune system, leading to inflammation that affects the brain. Research suggests that systemic inflammation triggers neuroinflammation, which can be detrimental and cause brain injury (Marshall, 2023). Then, brain pathologies associated with COVID-19 infection are likely to bring long-term impacts on cognitive processes (Kumar et al., 2021).

The relevant research traced the impacts of the prolonged COVID-19 process on mental health among students (Jang et al., 2023); nevertheless, the exclusive effects of the pandemic on the young have not been fully elucidated yet. Despite not being at the center of the pandemic, children and adolescents are deemed the riskiest groups to be hit by the adverse effects of the pandemic (O’sullivan et al., 2021). Unfortunately, children have had to adapt to unexpected changes in their daily lives due to the closure of educational institutions and libraries, the transition to distance education, and the emergence of the new normal. Indeed, they have had to bear the stress of being overwhelmed at home since having difficulties finding means of socialization (OECD, 2020a,b). Children who had COVID-19 exhibited higher rates of problems across various domains, including family, school, social, financial, and parental issues, as a result of the pandemic. Additionally, these children were found to be at a higher risk of experiencing multiple psychological problems, such as withdrawal, anxiety/depression, somatic problems, internalizing problems, externalizing problems, and total problems. Notably, a family history of psychiatric disorder and having three or more siblings were identified as high-risk factors for internalizing problems. While, the experience of school-related issues during the pandemic increased vulnerability to both internalizing and total problems (Ahmed et al., 2021). In the context of preschool children infected with COVID-19, they were more prone to encountering psychological issues, encompassing affective disorders, anxiety problems, pervasive developmental problems, and oppositional defiant problems. Importantly, a stronger association was observed between higher levels of anxiety and somatic symptoms and the negative impact of the pandemic on children’s lives (Ahmed et al., 2022). Overall, the pandemic has disrupted children’s routines, social interactions, and education, leading to elevated stress, anxiety, and depression (Huang and Zhao, 2020; Lakhan et al., 2020; Qiu et al., 2020; Jang et al., 2023).

Children who had a family history of psychiatric disorders and came from larger families (with three or more siblings) were more likely to experience internalizing problems. On the other hand, children who faced difficulties in school during the pandemic were more prone to both internalizing problems and overall problems.

Among them, students preparing for university admission exams have probably been the most affected group since they have had to deal with pandemic-related psychological challenges augmented by test anxiety (Yavas Celik, 2021). Counted among the obstacles to academic achievement, test anxiety concerns masses in Turkey (Baltas and Baltas, 2004) since Turkish high schoolers often experience it persistently when preparing for the university admission exam (YKS) for one or two years (Feta, 2019). Besides, it can be asserted that exam-preparing adolescents may have experienced such anxiety excessively due to the pandemic-related psychological distress and difficulties (Karatas, 2020). In this regard, the scholarly interest may be shifted to the role of mental well-being toward the stressors (e.g., test-anxiety) during tough times (e.g., pandemic).

With the emergent indicators of inner peace, happiness, self-esteem, self-confidence, and a sense of commitment, mental well-being is often conceived of as one’s ability to reveal their potential, cope with daily stress and difficulties, work efficiently, and contribute to their community (Keyes et al., 2002; WHO, 2019). It addresses conditions that may affect one’s physical, mental, and social health through past or present experiences (Vaingankar et al., 2011). Individuals with positive mental well-being always exert efforts to attain their goals, accept the ups and downs in life, and demonstrate resilience with the help of skills to cope with difficulties (Demirtas and Baytemir, 2019). Since individuals predisposed to psychological disorders may experience higher anxiety (Bagav, 2018), it is prudent to assert a possible relationship between one’s mental well-being and test anxiety. The relevant literature host research exploring psychological projections among students preparing for exams and the impacts of the exam preparation process on burnout, anxiety, mood, and emotional reactions (Esposito et al., 2020; Guessoum et al., 2020; Van De Groep et al., 2020; Gazmararian et al., 2021; Green et al., 2021; Jones et al., 2021; Kang et al., 2021; O’sullivan et al., 2021; Qin et al., 2021; Tang et al., 2021). Nevertheless, the research interest seems to have missed the relationship between mental well-being and test anxiety among students, even in times of the COVID-19 pandemic. Ultimately, the present study attempted to explore the link between test anxiety and mental well-being among senior high school students during the pandemic.

## 2. Materials and methods

### 2.1. Research design and sample

While the target population of this correlational study consisted of 929 high schooler seniors in Caycuma district of Zonguldak, Turkey in the 2020–2021 academic year, we selected 427 students as our sample using the snowball sampling technique. In this technique, a reference person is selected regarding the subject of the study, and others are reached through this person. The process is necessarily iterative. Participants lead researchers in growing the sample. Thus, it is referred to as the “snowball effect” (Yagar and Dokme, 2018). Accordingly, we asked the participating students to direct the online research link to their peers. In this study, we collected all the data online through Google Forms.

Among the participating students, 68.1% were girls, 57.6% of their mothers had primary school education, and 37.7% of their fathers held a high school diploma. The majority (78.7%) had their own room at home, and 53.2% had a personal computer (PC). While about half of the students (59.7%) thought that distance education failed, 68.9% could not be motivated for online classes during the pandemic.

### 2.2. Data collection tools

We collected the data using a demographic information form, the Warwick-Edinburgh Mental Well-Being Scale (WEMWBS), and the Westside Test Anxiety Scale (WTAS).

#### 2.2.1. Demographic information form

The form covers inquiries regarding gender, parental education, availability of a personal room and PC, perceived effectiveness of distance education, and the students’ motivation for online classes.

#### 2.2.2. Westside Test Anxiety Scale

The scale was developed by Driscoll (2007) to identify students with anxiety disorders and to promote intervention programs to alleviate their anxiety. Totan (2018) adapted the scale to the Turkish context. The 5-point Likert-type scale (1 = Never True, 5 = Always True) consists of 11 items. There is no reverse-coded item on the scale. Higher scores on the scale indicate higher test anxiety levels. The results of the Kaiser-Meyer-Olkin test (0.91) and Bartlett’s sphericity test (*χ^2^* = 1247.47, *SD* = 55, *p* = 0.000) revealed that the scale could sufficiently measure test anxiety among students. Finally, we calculated the internal consistency coefficient of the WTAS to be 0.91.

#### 2.2.3. Warwick-Edinburgh Mental Well-Being Scale

Tennant et al. (2007) introduced the scale to evaluate the mental well-being of individuals in England. The Turkish adaptation of the scale was carried out by Keldal (2015) with participants aged 16 years and older. The 5-point Likert-type scale (1 = Strongly Disagree, 5 = Strongly Agree) consists of 14 items within a single factor. Higher scores on the scale indicate “robust” mental well-being. While Cronbach’s alpha coefficient was calculated to be 89 in the original study, we found it to be 0.92.

### 2.3. Data collection procedure

We utilized Google Forms to create a questionnaire booklet for collecting data and employed the snowball sampling technique to reach out to participants between April 5 and May 12, 2021. We also informed the participants about the research through an informative text and obtained their informed consent. It took approximately 20 min to fill out the questionnaire booklet.

### 2.4. Limitations

Our findings are limited to the responses to the data collection tools by senior high school seniors in Caycuma district of Zonguldak, Turkey between April–May 2021.

### 2.5. Ethical considerations

We requested a conditional use permit from the corresponding authors for the WEMWBS and the WTAS. In addition, the Ethics Committee of Gumushane University granted ethical approval to our study (2021/3 dated 04.14.2021). Finally, we obtained informed consent from all the participating students.

### 2.6. Data analysis

While presenting continuous variables as means (*M*) and standard deviations (*SD*), we demonstrate categorical variables as numbers (*n*) and percentages (%). Prior to the data analysis, we resorted to the skewness and kurtosis values to check whether the data showed a normal distribution. In this sense, the data with skewness and kurtosis values between +1.00 and − 1.00 were regarded to distribute normally (Kim, 2013). Accordingly, we ran an independent samples t-test and one-way analysis of variance (ANOVA) to make group comparisons. Moreover, we performed Pearson’s correlation analysis to identify the direction and strength of the relationship between the research variables. All analyses were performed on the SPSS program, and we considered a *value of p* <0.05 statistically significant.

## 3. Results

As shown in Table 1, test anxiety significantly differed by gender [*t*_(425)_ = 2.16, *p* < 0.05]. Accordingly, we discovered the mean test anxiety score of the girls (*M* = 38.26) to be higher than that of the boys (*M* = 35.91). Yet, the participants’ mental well-being scores did not significantly differ by gender [*t*_(425)_ = −0.928, *p* > 0.05]. While mental well-being scores significantly differed between the participating students by maternal education (*F* = 3.110; *p* < 0.05), it was not the case for their test anxiety scores (*F* = 0.423; *p* > 0.05). The students with high school graduate mothers had significantly higher mental well-being scores than their peers with mothers holding an undergraduate degree. By paternal education, the participants significantly differed in test anxiety (*F* = 3.186; *p* < 0.05) but not in mental well-being (*F* = 0.792; *p* > 0.05). The mean test anxiety score was found to be significantly higher in the students with middle school graduate fathers than those with primary school graduate fathers (Table 2).

**Table 1:** Comparison of test anxiety and mental well-being scores by gender.

Scale	Gender	*n*	*M*	*SD*	*df*	*t*	*p*
WTAS	Female	291	38.26	10.33	425	2.16	**0.031**
	Male	136	35.91	10.81			
WEMWBS	Female	291	43.92	11.2	425	0.928	0.354
	Male	136	45.22	14.43			

Statistically significant values (*p*<0.05) are shown in bold.

**Table 2:** Comparison of test anxiety and mental well-being scores by parental education.

Scale	Maternal Education	*n*	*M*	*SD*	*df*	*F*	*p*	Difference
WTAS	Literate	8	36.5	11.35	426	0.423	0.792	–
	Primary School	246	37.27	10.73				
	Middle School	79	38.13	9.65				
	High School	70	38.43	10.48				
	Higher Education	24	35.71	11,76				
WEMWBS	Literate	8	48.38	15.14	426	3.11	**0.015**	**4–5**
	Primary School	246	44.61	11.81				
	Middle School	79	42.46	12.42				
	High School	70	47.13	13.12				
	Higher Education	24	38.29	11.64				

Scale	Paternal Education	*n*	*M*	*SD*	*df*	*F*	*p*	Difference
WTAS	Primary School	129	36.29	11.37	426	3.186	**0.024**	1–2
	Middle School	77	40.75	9.61				
	High School	161	37.22	9.47				
	Higher Education	60	36.82	11.89				
WEMWBS	Primary School	129	45.5	12.36	426	0.792	0.499	–
	Middle School	77	43.43	12.78				
	High School	161	44.39	11.69				
	Higher Education	60	42.9	13.31				

Statistically significant values (*p*<0.05) are shown in bold.

Our findings revealed that the students significantly differed in test anxiety scores by the availability of a personal room [*t*_(425)_ = 2.264, *p* < 0.05] and PC [*t*_(425)_ = 2.064, *p* < 0.05]. Accordingly, the participants without a PC and personal room had higher test anxiety scores. Nevertheless, their mental well-being scores did not differ significantly by said variables [*t*_(425)_ = 0.660 vs. −0.184, *p* > 0.05; Table 3].

**Table 3:** Comparison of test anxiety and mental well-being scores by availability of a personal room and PC.

Scale	Personal Room	*n*	*M*	*SD*	*df*	*t*	*p*
WTAS	Yes	336	36.91	10.45	425	2.264	**0.024**
	No	91	39.72	10.62			
WEMWBS	Yes	336	44.13	12.11	425	0.66	0.509
	No	91	45.09	13.08			

Scale	PC	*n*	*M*	*SD*	*df*	*t*	*p*
WTAS	Yes	227	36.53	10.82	425	2.064	**0.04**
	No	200	38.63	10.11			
WEMWBS	Yes	227	44.44	12.99	425	−0.184	0.854
	No	200	44.25	11.53			

Statistically significant values (*p*<0.05) are shown in bold.

While the students’ mental well-being scores significantly differed by perceived effectiveness of distance education (*F* = 3.945; *p* < 0.05), it was not the case for their test anxiety scores (*F* = 1.388; *p* > 0.05). The students perceiving distance education as an effective method had higher mental well-being scores. On the other hand, the participants’ test anxiety and mental well-being scores significantly differed by motivation for online classes (*F* = 5.529 vs. 11.903; *p* < 0.05). Accordingly, the students thinking being always and sometimes motivated for online classes had lower test anxiety scores than others. Conversely, those perceiving no motivation for online classes had lower mental well-being scores than their peers (Table 4).

**Table 4:** Comparison of test anxiety and mental well-being scores by perceived effectiveness of distance education and motivation for online classes.

Scale	Perceived Effectiveness of Distance Education	*n*	*M*	*SD*	*df*	*F*	*p*	Difference
WTAS	Always	25	36.04	11.68	426	1.388	0.251	**–**
	Never	255	38.2	10.59				
	Sometimes	147	36.57	10.21				
WEMWBS	Always	25	48.88	14.36	426	3.945	**0.02**	1–2
	Never	255	43.1	12.02				3–2
	Sometimes	147	45.71	12.22				

Scale	Motivation for Online Classes	*n*	*M*	*SD*	*df*	*F*	*p*	Difference
WTAS	Always	22	35.09	10.71	426	5.529	**0.004**	3–2
	Never	294	38.64	10.35				
	Sometimes	111	35	10.57				
WEMWBS	Always	22	50.68	15.45	426	11.903	**0.001**	1–2
	Never	294	42.46	11.98				3–2
	Sometimes	111	48.04	11.33				

Statistically significant values (p<0.05) are shown in bold.

Finally, the findings showed a low, negative significant relationship between test anxiety and mental well-being, implying that test anxiety among the students decreases as their mental well-being levels increase (Buyukozturk et al., 2020; Table 5).

**Table 5:** Correlation between mental-well being and test anxiety scores.

		WTAS	WEMWBS
WTAS	*r*	1	−0.285
	*p*		**0.000**
	*n*	427	427
WEMWBS	*r*	−0.285	1
	*p*	**0.000**	
	*n*	427	427

**Significant at 0.01. Statistically significant values (*p*<0.05) are shown in bold.

## 4. Discussion

In this study, we explored any potential association between test anxiety and mental well-being among high school students preparing for the university admission exam in times of the pandemic. The findings revealed a significant difference in the participants’ test anxiety by gender. Despite a plethora of research demonstrating higher test anxiety among girls than boys (Alyaprak, 2006; Bonaccio and Reeve, 2010; Serim, 2016), Isikay (2019) concluded vice versa. Turkish girls may desire more independence and a successful career against societal expectations since such outdated expectations are known to oriented to hinder women’s role in society in Turkey. As an illustration, when it comes to the intersection of academic and career achievements, Turkish academia boasts of a relatively balanced gender representation with 46.2% of female academicians among the entire academic workforce (YOK, 2023). Additionally, it has a higher proportion of female professors compared to Australia (Ozkanlı and White, 2008). Thus, girls consider having a successful career a solution to avoid traditional social role perceptions, which may explain higher test anxiety levels among our participating girl students. However, we could not concşude a significant difference between gender and mean mental well-being, overlapping with previous findings (Ekti, 2019; Sahin, 2019; Ozyurt, 2020). It should be noted that some other studies revealed that male (Hori, 2010; Khan et al., 2015) or female students (Ryff, 1989; Kuyumcu, 2012; Ayhan, 2020) have higher mental well-being levels. Cultural and socioeconomic differences may account for mixed results in the literature.

We found out that the students’ mental well-being significantly differed by maternal education, while it was not the case by paternal education. Interestingly, the students with illiterate mothers demonstrated higher mental well-being levels than those with educated mothers. In the study by Hamurcu (2011), maternal education showed significant differences in the positive relationships, purpose in life, and self-acceptance dimensions of psychological well-being, while there were significant differences between paternal education and the autonomy and individual development dimensions. Besides, some studies showed parental education does not have a substantial impact on adolescents’ psychological well-being (Karabeyeser, 2013; Altuntas, 2018). An educated mother may expect exceptional performance from her child than their capabilities, which may adversely affect their mental well-being.

We determined test anxiety among the participants significantly differed by paternal education. Thus, the students with middle school graduate fathers had higher test anxiety levels than their peers with fathers with primary school education. Similar to our finding above, an educated father may expect high performance from his child, contributing to the student’s test anxiety. The literature, on the other hand, offers contradictory results; while some reported that higher educational attainment of fathers is associated with decreased test anxiety among students (Alyaprak, 2006; Zengin, 2019), there are studies reporting that paternal education is not linked with children’s test anxiety (Kacan Softa et al., 2015; Karacor, 2020).

Non-availability of a personal room and PC was found to be a factor contributing to an increase in the students’ test anxiety. Given the compulsory nature of distance education due to the pandemic, being deprived of such opportunities may have caused the participants to be distracted by excessive stimuli (TV, sounds, outside noise, etc.), leading to increased test anxiety. Kirali and Alci (2016) found that undergraduate students owning a PC had a more positive perception of distance education than their peers without a PC at home. Nevertheless, substantial evidence showed no significant association between study environment test anxiety (Kayapinar, 2006; Duman, 2008; Dundar, 2018; Bilir, 2019).

More than half of the participants (59.7%) considered distance education ineffective, and we found that the students finding distance education to be an effective learning method had higher mental well-being levels. Our results overlap what was previously found in the literature (Baris, 2015; Atabey, 2016; Kirali and Alci, 2016; Yavuz, 2016). Therefore, we can confidently propose that the students favored face-to-face education, but distance education had no effect on test anxiety. In addition, our findings imply that students acknowledging distance education as an effective learning method have better outcomes in lessons and feel better psychologically during classes.

Being always and sometimes motivated for online classes seemed to be linked with lower test anxiety levels among the participants. Their mental well-being levels also significantly differed by motivation for online classes; those not motivated for online classes had lower mental well-being levels. These results imply that the non-motivated students are not likely to follow the lessons and, thus, may think that they will fail exams, which may adversely affect their mental well-being. In their study on students’ perspectives on distance education, Metin et al. (2017) found that the participants did not believe in the effectiveness of distance education and thought they would be more successful in face-to-face classes. Similarly, Sercemeli and Kurnaz (2020) concluded that students did not think that they could academically capitalize on distance education and were not motivated for their classes during the pandemic.

Finally, we found a low negative relationship between students’ test anxiety and mental well-being levels. The research with high school and undergraduate students (López et al., 2013; Mckay and Andretta, 2017) reported mental well-being to be positively linked with positive emotions, life satisfaction, and physical health but negatively related to psychosomatic symptoms. In addition, some scholars emphasized that individuals with “robust” mental well-being are less predisposed to physical and mental disorders and utilize healthcare services less (Keyes, 2005; Nordentoft, 2007). Besides, text anxiety was previously found to be associated with depression, subjective well-being, and perceived stress levels (Abbasoglu, 2018; Bagav, 2018; Isikay, 2019; Baspinar Erten, 2020). Ultimately, one with high test anxiety may have elevated stress and become depressed, which, in turn, undermines their mental well-being. Such individuals are likely to develop negative thoughts about themselves and may not enjoy life.

## 5. Conclusion

Overall, gender, paternal education, availability of a personal room and PC, and motivation for online classes were found to be the factors contributing to test anxiety. We also found the factors that affect mental well-being among students were parental age, maternal education and employment, the device used for online classes, perceived effectiveness of distance education, and motivation for online classes. Besides, our analysis revealed a low, negative significant association between the participants’ test anxiety and mental well-being levels.

In line with our findings, we may offer the following recommendations to experts, families, and researchers:

- Informative seminars may be held to reduce female students’ test anxiety levels during exam preparation.
- Parents may be informed about facilitating approaches and attitudes toward their children. Teachers and field experts may collaborate to organize guiding seminars for parents on exam preparation process.
- Field experts may inform teachers and parents about students’ mental well-being.
- Teachers and relevant education professionals may organize activities to boost students’ motivation for online classes.
- Families may be informed about the significance of positive interaction with their children and its impact on children and adolescents.
- Social campaigns can be initiated to raise awareness of the impacts of test anxiety on students’ well-being and provide strategies to manage and reduce anxiety.
- Further research may trace the relationship between test anxiety and mental well-being levels of senior middle and high school students in different cities and districts.
- Further research may also focus on the relationships between mental well-being and some socio-demographic variables of different age groups.
- Prospective researchers may engage in longitudinal research to investigate the long-term effects of test anxiety on students’ well-being and academic performance, considering societal perceptions of gender and parental influence.

## Data availability statement

The original contributions presented in the study are included in the article/supplementary material, further inquiries can be directed to the corresponding author.

## Ethics statement

The studies involving human participants were reviewed and approved by Gumushane University Ethics Committee (Decision date 14.04.2021, decision number: 2021/3). Written informed consent to participate in this study was provided by the participants’ legal guardian/next of kin.

## Author contributions

NA, LCG, and ST contributed to data interpretation and critical review. EY were responsible for the collection of data and literature review. EY, LCG, and ST were in charge for literature review. NA and LCG was in charge for study design and essential help. All authors contributed to the study conception, design, analyzed and interpreted data, drafted the article, and approved the submitted version.

## Conflict of interest

The authors declare that the research was conducted in the absence of any commercial or financial relationships that could be construed as a potential conflict of interest.

## Publisher’s note

All claims expressed in this article are solely those of the authors and do not necessarily represent those of their affiliated organizations, or those of the publisher, the editors and the reviewers. Any product that may be evaluated in this article, or claim that may be made by its manufacturer, is not guaranteed or endorsed by the publisher.
